# Supplementary material for: Dietary palaeoecology of an Early Cretaceous armoured dinosaur (Ornithischia; Nodosauridae) based on floral analysis of stomach contents
Source: R Soc Open Sci. 2020 Jun 3;7(6):200305. doi: 10.1098/rsos.200305 (PMC7353971; doi:10.1098/rsos.200305)
Supplement: Electronic Supplementary Material [file rsos200305supp2.pdf]

Supplemental Material for: Dietary palaeoecology of an Early Cretaceous armoured dinosaur (Ornithischia; Nodosauridae) based on floral analysis of stomach contents

Caleb M. Brown[1], David R. Greenwood[2], Jessica E. Kalyniuk[2], Dennis R. Braman[1], Donald M. Henderson[1], Cathy L. Greenwood[2], James F. Basinger[3]

[1] Royal Tyrrell Museum of Palaeontology, Drumheller, Alberta, Canada T0J 0Y0; [2] Department of Biology, Brandon University, Brandon, Manitoba, Canada R7A 6A9; [3] Department of Geological Sciences, University of Saskatchewan, Saskatoon, Saskatchewan, Canada S7N 5E2

1. Supplemental Materials and Methods

1.1 Macroscopic analysis of abdominal mass ('cololite')

Examination and photography of the abdominal mass (the cololite) was conducted under ambient light as well as cross-polarized light and ultraviolet light (UV) fluorescence. Cross-polarized light was used in combination with wetting of the rock surface with water to bring about additional contrast. The majority of the volume of the mass is contained within four large body blocks (Block D, E, F, and K), with additional limited material on a fifth block (counter slab of F) – See Brown [1]. To estimate the volume and three-dimensional form of the entire abdominal mass, exposed cross sections were photographed and measured. Three large hand samples of exclusively abdominal mass material (782, 235, and 205 g), which fit together and fit onto the dorsal surface of block K, were scanned using computed tomography, and documented photogrammetrically, to allow for digital dissection and volume estimation. Numerous smaller hand samples of uncertain position and orientation within the mass were also documented and several used for histological thin sections.

An estimate of the volume of the cololite and its reconstruction were done with measurements taken from the *in situ* material that was visible on the main blocks. The formula for the volume of a ellipsoid is given by $(4/3)\pi abc$, where 'a' is the major radius, 'b' is the intermediate radius, and 'c' is the semi-minor radius.

Anteroposteriorly, the cololite is located near the thoracosacral transition, in line with the ninth through twelfth transverse thoracic osteoderm rows [1] (figure 1, 2), in the same transverse plane as the anteriormost margin of the ilium farther laterally. The cololite is located entirely to the left of the midline (minimum known distance from midline = 5.5 cm, maximum known distance from midline = 30 cm, distance from centre to midline = 19.5 cm). Dorsoventrally, the cololite is located far dorsally such that its dorsal extent encloses the ventral margin of the ribs in the T11–12 rows, and is nearly appressed to the osteoderms of rows T11–12 [1] (figure 2*d*–*f*). Although anatomically the dorsal position represents up, the animal came to rest on its back, and the dorsal position is stratigraphically down, and represents settling of the viscera. The cololite does not appear to extend posterior to T12, but does extend anteriorly to T9 (into block D and E) and medially almost to the midline (into Block F). The lateral extreme of the cololite was lost in the discovery phase, and the abrupt margin indicates that the mass also extended a significant amount laterally, but the extent of this is unknown. Based on the occurrence of the cololite and the nature of the boundaries, the preserved extent can be documented, and the original size estimated. Given the curved posterior boundary in dorsal view (block K), and the extent observable in cross section (blocks D, E and F) the preserved shape is roughly a vertically compressed sphere (oblate spheroid). In dorsal view, the parasagittal, oblique, and transverse, diameters are 36.5 cm, 37 cm, and 32 cm, respectively, with the first two measures complete and the final measure truncated laterally. The vertical height of the cololite varies between 8.0 cm posteriorly, 8.5 cm anteriorly and laterally, and 12.5 cm more centrally. Given these observed mass dimensions, mean values for the ellipsoid formula were: *a*; 18.3 cm, *b*; 14.5 cm, and *c*; 6.25 cm, with the calculated volume of the collapsed mass 6.93 L.

The margin of the cololite is observable in several cross-sections including: dorsal margin in two parasagittal sections; ventral margin in two parasagittal sections; posterior margin in horizontal section; and anterior margin in several oblique vertical sections (figure 1, S1). In addition, several hand samples

(from unknown locations) also preserved the margin of the cololite, either as a cross section, or as a cleavage plane parallel to the mass margin.

1.2 Microscopic palaeobotanical analysis of cololite

Initial analysis applied a systematic survey using a transect system, commencing at the left top corner of the slide, and proceeding across the slide in successive transects. At each new field of view, using the 10X objective, a numbered image was taken, and from the field of view every plant fragment seen was assigned to one of several categories to produce a tally for that field of view. Counting was done using the microscope's image software (cellSens™, Olympus) to divide the field of view on the computer screen into a 10 x 10 grid and recording the number of grid cells in which each plant fragment category occurred. Grid squares with gastroliths and matrix were also recorded. This comprehensive approach served as a quality control for the subsampling approach used for the remainder of the slides.

Subsequent analysis of four additional slides used a subsampling approach, where all possible transects for each slide were assigned a number, and then 10 transects were selected from these numbered transects for counting using a random number generator (random.org). For these transects, gastroliths and matrix were not individually scored for grid cells; however, a record was kept of the grid cells not scored for plant fragments, as no plant fragments (i.e., only gastroliths or matrix, or both, present). At the completion of the transect quantitative survey, the slides were visually scanned again to detect well-preserved examples of key plant fragment categories to produce an image catalogue for more refined taxonomic assessment. A total of five slides were counted, resulting in ~1000 individual fields of view counted, with each field of view including 100 individual grid squares. All data and the image collection are archived and available on request.

Taxonomic identifications, where possible, were made using available reference material of the leaf and young stem/petiole cross-section anatomy of fossil and extant ferns and horsetails (i.e., pteridophytes), gymnosperms such as cycads–cycadophytes, *Ginkgo* and conifers, and basal angiosperms, as well as the epidermal/cuticle anatomy of these plant groups [2-9]. For example, leaves of extant cycads, as typified by the species *Cycas circinalis*, have a multi-cell-thick layer of prominent sclerenchyma (thickened cells) – the hypodermis – underlying the leaf epidermis that is particularly well developed on, or restricted to, the leaf mid-vein in species such as *Zamia floridana*. Additionally, cycads commonly have stomata with polar flanges [e.g., 10, 11] and Bennettitales have diagnostic epidermal and stomatal patterns [9]. Similarly, conifer families implicated in the literature as possible food sources of dinosaurs [12-14], such as the Araucariaceae, Cheirolepidiaceae, Podocarpaceae and taxodioid Cupressaceae, have distinctive and often diagnostic epidermal/cuticular anatomy [2-5, 7].

2. Supplemental Results

Scoring of each criterion for TMP 2011.033.0001 (table S2 & S3).

Criterion (Score) - Justification

1 (1) - The fossil of the animal itself and the putative cololite with plant material are closely associated with each other, and far from their natural habitat (Co-allochthonous)

2 (1) - The putative cololite is located both inside the body cavity

3 (1) - and in the correct position for a stomach

4 (1) - The whole animal fossil shows exceptional preservation of other tissues

5 (1) - The organic inclusions within of the putative cololite are of small and similar size

6 (1) - The organic inclusions show distinct cut margins

7 (1) - The organic inclusions are associated with gastroliths

8 (1) - The putative cololite has a mineralogy distinct from that of the surrounding matrix

9 (1) - Distinct margins of the abdominal mass are observable in cross sections

10 (1) - The putative cololite has a three-dimensional spheroidal shape

11 (1) - The plant fragments found in the cololite are absent from the surrounding matrix

12 (1) - The cololite contains intact sporangia from a diversity of taxa representing an unusual concentration

13 (1) - The palynomorphs from within the cololite are a subset of the external sample

14 (NA) - Acid etching in bone is a carnivore criterion and not applicable

15 (?) - No geochemical analysis of the cololite was performed

16 (1) - The organic inclusions of the cololite are dietarily appropriate

References

1. Brown C.M. 2017 An exceptionally preserved armored dinosaur reveals the morphology and allometry of osteoderms and their horny epidermal coverings. *PeerJ* **5**, e4066.
2. Daghlian C.P., Person C.P. 1977 The cuticular anatomy of *Frenelopsis varians* from the Lower Cretaceous of central Texas. *American Journal of Botany* **64**(5), 564-569.
3. Stockey R.A. 1994 Mesozoic Araucariaceae: morphology and systematic relationships. *Journal of Plant Research* **107**(4), 493-502.
4. Leng Q., Yang H., Yang Q., Zhou J. 2001 Variation of cuticle micromorphology of *Metasequoia glyptostroboides* (Taxodiaceae). *Botanical Journal of the Linnean Society* **136**(2), 207-219.
5. Ma Q.-W., Li C.-S. 2002 Epidermal structures of *Sequoia sempervirens* (D. Don) Endl.(Taxodiaceae). *Taiwania* **47**(3), 194-202.
6. Ma Q.-W., Li C.-S., Li F.-L., Vickulin S.V. 2004 Epidermal structures and stomatal parameters of Chinese endemic *Glyptostrobus pensilis* (Taxodiaceae). *Botanical journal of the Linnean Society* **146**(2), 153-162.
7. Greenwood D.R., Hill C.R., Conran J.G. 2013 *Prumnopitys anglica* sp. nov. (Podocarpaceae) from the Eocene of England. *Taxon* **62**(3), 565-580.
8. CUPAC. 2018 Cornell University Plant Anatomy Collection, online. http://cupac.bh.cornell.edu/ [accessed November 2018].
9. Rudal P.J., Bateman R.M. 2019 Leaf surface development and the plant fossil record: stomatal patterning in Bennettitales. *Biological Reviews* **94**(3), 1179-1194.
10. Passalia M.G., Del Fueyo G., Archangelsky S. 2010 An Early Cretaceous zamiaceous cycad of south west Gondwana: *Restrepophyllum* nov. gen. from Patagonia, Argentina. *Review of Palaeobotany and Palynology* **161**(3-4), 137-150.
11. Su K., Quan C., Liu Y.-S.C. 2014 *Cycas fushunensis* sp. nov.(Cycadaceae) from the Eocene of northeast China. *Review of Palaeobotany and Palynology* **204**, 43-49.
12. Sander P.M., Gee C.T., Hummel J., Clauss M. 2010 Mesozoic plants and dinosaur herbivory. In *Plants in Mesozoic Time: morphological innovations, phylogeny, ecosystems* (ed. Gee C.T.), pp. 331-359. Bloomington, Indiana University Press.
13. Mallon J.C., Anderson J.S. 2014 The functional and palaeoecological implications of tooth morphology and wear for the megaherbivorous dinosaurs from the Dinosaur Park Formation (upper Campanian) of Alberta, Canada. *PloS one* **9**(6), e98605.
14. Gee C.T. 2011 Dietary options for the sauropod dinosaurs from an integrated botanical and paleobotanical perspective. In *Biology of the sauropod dinosaurs: Understanding the life of giants* (eds. Klein N., Remes K., Gee C.T., Sander P.M.), pp. 34-56. Bloomington, Indiana University Press.
15. Raine J.I., Mildenhall D.C., Kennedy E.M. 2011 New Zealand fossil spores and pollen: an illustrated catalogue. 4th edition. In *GNS Science miscellaneous series no 4*
16. Jameossanaie A. 1987 Palynology and age of South Hospah coal-bearing deposits, McKinley County, New Mexico. *New Mexico Bureau of Mines & Mineral Resources* **Bulletin 112**, 66 p.
17. The Paleobiology database, online. https://paleobiodb.org [accessed January 2020]

TABLE S1: Evaluation of support for reported stomach contents based on the criteria of Table 2. Scores were based on published accounts (see Table 1) and specimens were not independently reexamined in this study.

**SPECIMEN**	**TMP 1980.040.0001**	**TMP 1990.104.0001**	**JRF 115H**	**QM F18101**	**MOZ-PV 6459**	**TMP 2011.033.0001**
CRITERION	***Corythosaurus casuarius***	**Hadrosauridae**	***Brachylophosaurus canadensis***	***Kunbarrasaurus ieversi***	***Isaberrysaura mollensis***	***Borealopelta markmitchelli***
**1**	0	0	0	1	0	1
**2**	1	1	1	1	1	1
**3**	1	1	1	1	1	1
**4**	1		1	1	0	1
**5**	1	NA	1	1	1	1
**6**	0	0	?	1	0	1
**7**	NA	NA	NA	0	0	1
**8**	0	0	1	1	?	1
**9**	0	0	0	0	?	1
**10**	0	0	0	1	1	1
**11**	1	1	1	1	1	1
**12**	0	1	0	0	1	1
**13**	?	?	0	?	?	1
**14**	NA	NA	NA	NA	NA	NA
**15**	?	?	?	?	?	?
**16**	1	0	1	1	1	1
**TOTAL**	**6**	**4**	**7**	**10**	**7**	**14**
	**Equivocal**	**Not supported**	**Equivocal**	**Strong**	**Strong**	**Unequivocal**

TABLE S2: Percentage values for the grouped categories of plant fragments (all slide counts summed). Values are rounded to whole numbers in the main text.

Plant Fragment Major Group	Abundance (%)
**Leaf Tissue**	**87.6**
Leaf epidermis with cellular material	1.9
Leaf cross-section (incl. clumps of thickened cells/sclerenchyma)	8.5
Sporangia	4.3
Undifferentiated plant material (likely leaf mesophyll)	68.8
**Stems**	**6.7**
Wood and isolated tracheids	3.6
Stem cross-sections	3.1
**Charcoal / Blackened plant fragments**	**5.7**
**Total**	**100.0**

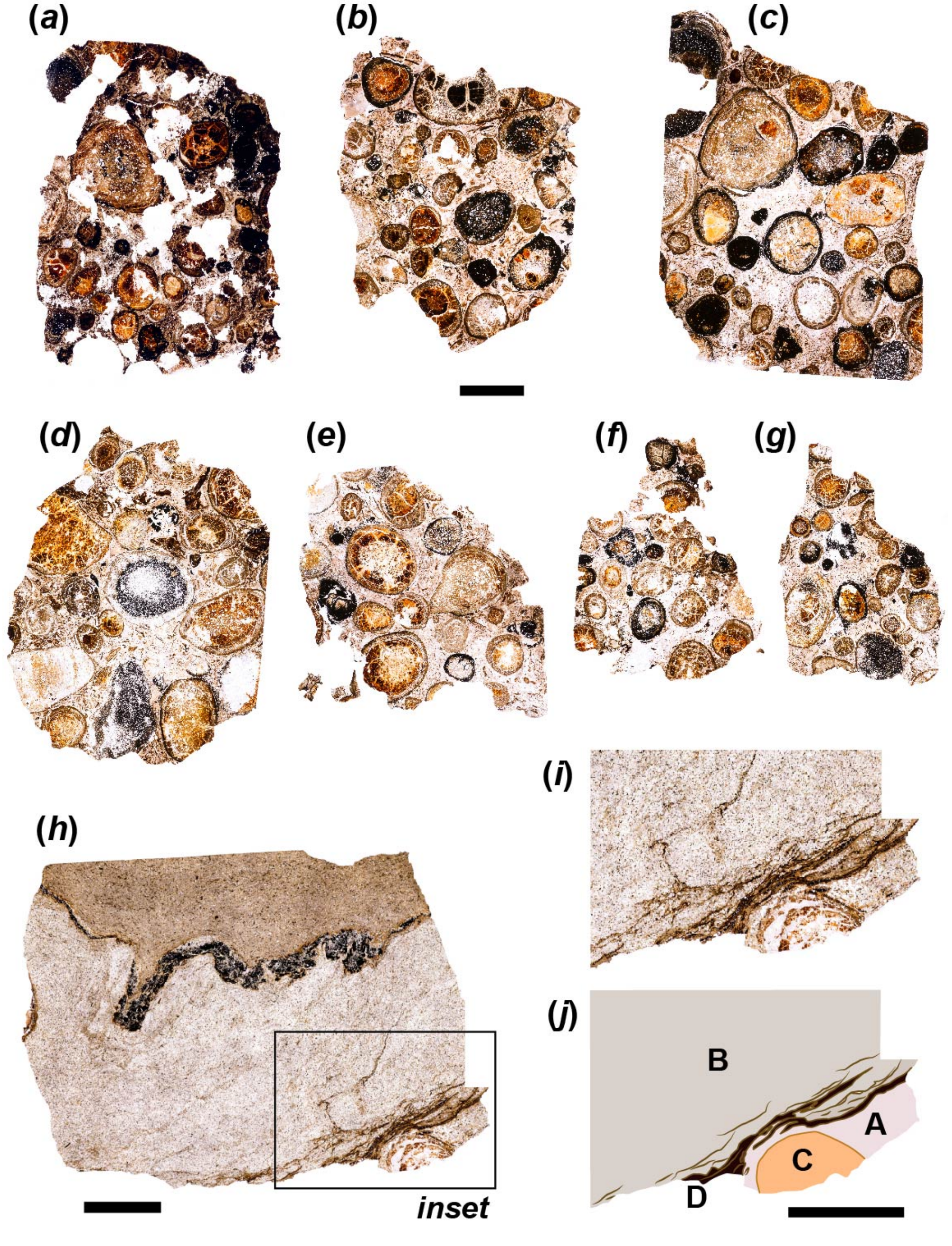

FIGURE S1: Photographs of histological thin sections of hand samples of the abdominal mass of TMP 2011.033.0001. (*a*) slide 1, (*b*) slide 2, (*c*) slide 3, (*d*) slide 4, (*e*) slide 5, (*f*) slide 6, (*g*) slide 7, (*h–j*) slide 8. (*i*) and (*j*) are photographic and interpretive insets of (*h*). Scale bars equals 1 cm. A: cololite matrix; B: external matrix; C: gastolith; D: cololite margin.

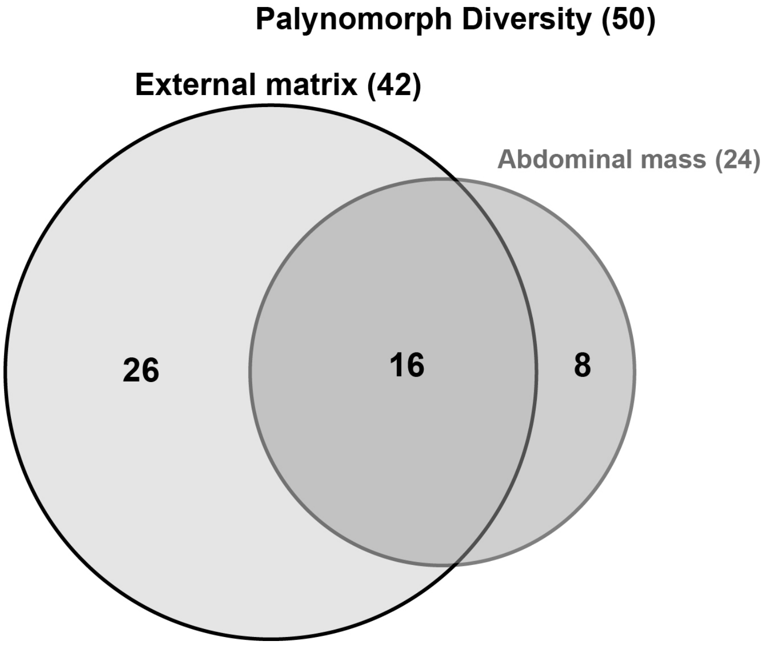

FIGURE S2: Venn diagram of palynomorph diversity in the entire sample (50), external matrix (42), and the abdominal mass (24). Circle radii scaled to diversity. See Appendix 1 for details.

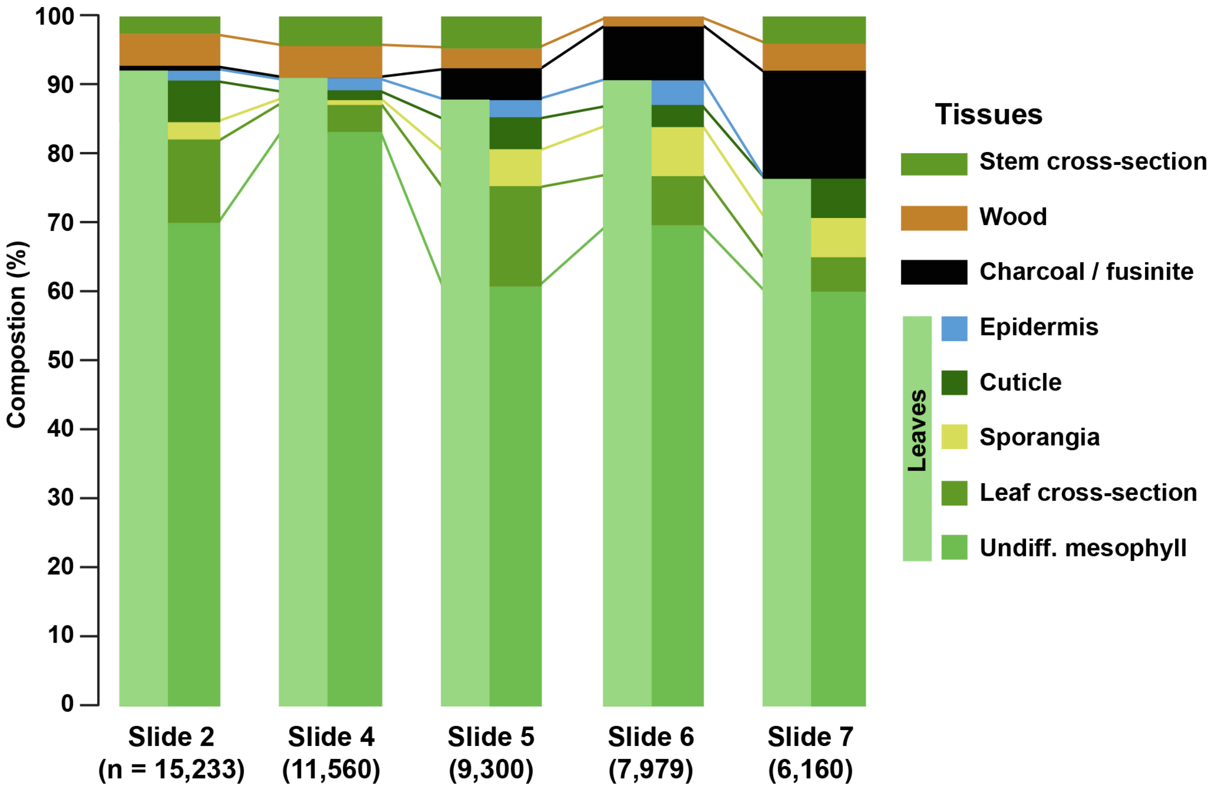

FIGURE S3: Breakdown of the plant fragment composition into tissue types, and leaf specific tissue types, across all five slides. Sample sizes, in brackets, indicates the number of grid squares counted for each slide (note that for slide 2 this number includes non-plant components).

Appendix 1:
Palynomorph species recovered from TMP 2011.033.0001. Palynological number: TMP 2017.205

PALYNOMORPH	HIGHER TAXONOMY [15-17]	COLOLITE ONLY	BOTH	EXTERNAL ONLY
**BRYOPHYTE AND PTERIDOPHYTE SPORES**				
Aequitriradites verrucosus (Cookson & Dettmann) Cookson & Dettmann 1961	Bryophyta s.l.			1
Baculatisporites comaumensis (Cookson) Potonié 1956	Polypodiidae: Osmundaceae		1	
Baculatisporites sp.	"		1	
Biretisporites deltoidus (Rouse) Dettmann 1963	Polypodiidae: Schizaeaceae	1		
Cibotiumspora juncta (Kara-Murza) Singh 1983	Polypodiidae: Cibotiaceae-Dicksoniaceae		1	
Cicatricosisporites augustus Singh 1971	Polypodiidae: Schizaeaceae			1
Cicatricosisporites hughesii Dettmann 1963	"		1	
Cicatricosisporites sp.	"		1	
Cingutriletes sp.	Bryophyta s.s.			1
Cyathidites australis Couper 1953	Polypodiidae: Cyatheaceae or Dicksoniaceae or Lygodiaceae		1	
Cyathidites australis rimalis Balme 1957	"			1
Cyathidites minor Couper 1953	"		1	
Deltoidospora diaphana Wilson & Webster 1946	Polypodiidae: indet.	1		
Distaltriangulisporites irregularis Singh 1971	Polypodiidae: Schizaeaceae			1
Distaltriangulisporites perplexus (Singh) Singh 1971	"			1
Echinatisporis sp,	Lycopodiopsida: Lycopodiaceae or Selaginellaceae	1		
Foraminisporis asymmetricus (Cookson & Dettmann) Dettmann 1963	Bryophyta s.s.			1
Foraminisporis sp.	"			1
Gleicheniidites circinidites (Cookson) Krutzsch 1959	Polypodiidae: Gleicheniaceae			1
Gleicheniidites delicatus (Bolkhovitina) Krutzsch 1959	"		1	
Gleicheniidites senonicus Ross 1949	"		1	
Gleicheniidites stellatus (Bolkhovitina) Krutzsch 1959	"			1
Impardecispora apiverrucata (Couper) Venkatachala, Kar & Raza 1969	Polypodiidae: indet.			1
Impardecispora canadensis (Pocock) Venkatachala, Kar & Raza 1969	"			1
Impardecispora trioreticulosus (Cookson & Dettmann) Venkatachala 1969	"			1
Interulobites sp.	Bryophyta s.l.			1
Klukisporites pseudoreticulatus Couper 1958	Polypodiidae: Schizeaceae		1	
Kraeuselisporites sp.	Lycopodiopsida			1
Laevigatosporites haardti Wilson & Webster 1946	Polypodiidae: indet.		1	
Osmundacidites wellmanii Couper 1953	Polypodiidae: Osmundaceae	1		
Retitriletes singhii Srivastava 1972	Lycopodiopsida			1
Stereisporites antiquasporites (Wilson & Webster) Dettmann 1963	Bryophyta s.s.		1	
Tigrisporites scurrandus Norris 1967	Polypodiidae: indet.			1
Todisporites minor Couper 1958	Polypodiidae: ?Osmundaceae			1
**PTERIDOSPERM AND GYMNOSPERM POLLEN**				
Alisporites bilateralis Rouse 1959	pteridosperm		1	
Alisporites grandis (Cookson) Dettmann 1963	"			1
Alisporites sp.	"			1

Classopollis classoides Pflug 1953	Pinales: Cheirolepidiaceae			1
Cycadopites formosus Singh 1964	Cycadales: Fam. indet.			1
Pityosporites constrictus Singh 1964	Pinales: Pinaceae	1		
Pityosporites sp.	"	1		
Podocarpidites multesimus (Bolkhovitina) Pocock 1962	Cupressales: cf. Podocarpaceae			1
Pristinuspollenites microsaccus (Couper) Tschudy 1973	Caytoniales			1
Taxodiaceaepollenites hiatus (Potonié) Kremp 1949	Cupressales: 'taxodioid' Cupressaceae			1
Taxodiaceaepollenites vacuipites (Wodehouse) Wingate 1980	"		1	
Tsugaepollenites mesozoicus Couper 1958	Pinales: Pinaceae cf. *Tsuga*			1
Vitreisporites pallidus (Reissinger) Nilsson 1958	Caytoniales: cf. *Sagenopteris*		1	
**ANGIOSPERM POLLEN**				
Tricolpites sp.	Indet.	1		
Tricolporites sp.	Indet.	1		
**DINOFLAGELLATES**			1	
**TOTAL**		**8**	**16**	**26**
